# Identification of SARS CoV‐2 Omicron BA.1 and a novel Delta lineage by rapid methods and partial spike protein sequences in Sulaymaniyah Province, Iraq

**DOI:** 10.1002/iid3.801

**Published:** 2023-03-17

**Authors:** Mariwan Kadir Rasheed, Harem Abdalla Awrahman, Sirwan M. Amin Al‐Jaf, Sherko S. Niranji

**Affiliations:** ^1^ College of Health Science University of Human Development Sulaymaniyah Iraq; ^2^ Sulaimani Veterinary Directorate Sulaimani Iraq; ^3^ Hiwa Hospital, Sulaymaniyah General Directory of Health Ministry of Health Sulaymaniyah Iraq; ^4^ College of Medicine University of Garmian Kalar Iraq; ^5^ Coronavirus Research and Identification Lab University of Garmian Kalar Iraq

**Keywords:** Delta, Iraq, method, Omicron, SARS CoV‐2

## Abstract

**Background:**

Five variants of concern (VOCs) of severe acute respiratory syndrome coronavirus 2 (SARS CoV‐2) have been globally recorded including Alpha, Beta, Gamma, Delta, and Omicron. The Omicron variant has outcompeted the other variants including the Delta variant. Molecular screenings of VOCs are important for surveillance, treatment, and vaccination programs. This study aimed to identify VOCs by using rapid inexpensive methods and partial sequencing of the virus's spike gene.

**Methods:**

Mutation‐specific rRT PCR probes were used for both D614G and K417N mutations to potentially discriminate between Delta and Omicron variants. These were followed by sequencing of a fragment of spike gene (748 nucleotides), which covers the most notable VOC mutations in the receptor binding domain of SARS CoV‐2.

**Results:**

Rapid methods showed that out of 24 SARS CoV‐2 positive samples, 19 carried the N417 mutation, which is present in the Omicron variant. Furthermore, 3 samples carried K417 wildtype, which is present in the Delta variant. Additionally, 2 samples containing both K417 and N417 suggested mixed infections between the two variants. The D614G mutation was present in all samples. Among the 4 samples sequenced, 3 samples carried 13 mutations, which are present in Omicron BA.1. The fourth sample contained the two common mutations (T478K and L452R) present in Delta, in addition to two more rare mutations (F456L and F490S), which are not commonly seen in Delta. Our data suggested that both Omicron variant BA.1 and a novel Delta variant might have circulated in this region that needs further investigations.

## Graphical abstract

Investigationos of SARS CoV‐2 variants of concerns (VOCs) have been ignored in developing countries including Iraq. Spike gene sequencings and rapid methods were used to identify VVOCs. Current study identified the Omicron BA1 sub‐variant and a novel Delta variant in Sulaymaniah province of Iraq.

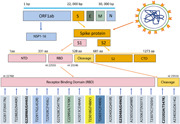

## 1 INTRODUCTION

Severe acute respiratory syndrome coronavirus‐2 (SARS CoV‐2), which is the causative agent for coronavirus 2019 (Covid‐19), has several structural proteins including Spike (S), Envelope (E), Membrane (M), and Nucleocapsid (N). The spike protein (1273 amino acids) has the receptor binding domain (RBD), which is important for interactions with the human angiotensin‐converting enzyme 2 receptor. Mutations in the spike gene are important for determining the variants of SARS CoV‐2 that impact on immune evasion ability of the virus leading to emerging variants of concern (VOCs).

The VOCs of SARS CoV‐2, which have notable mutations (ranging from 3 to 30 mutations) in its spike protein, have been globally identified that can be summarized according to the World Health Organization (WHO) label (Pango Lineage) as follows: Alpha (B.1.1.7), Beta (B.1.351), Gamma (P.1), Delta (B.1.617.2), and Omicron (B.529).^1^ The details of notable mutations are summarized in Table 1 as also described in references.^1^, ^2^

**Table 1:** Classifications of variants of concerns of SARS CoV‐2 according to WHO label and Pango lineage.

WHO label	Pango linage	First country	Date of detection	Notable mutations
Alpha	B.1.1.7	United Kingdom	September 2020	N501Y, A570D
Beta	B.1.351	South Africa	September 2020	N501Y, E484K, K417N
Gamma	P.1	Brazil	December 2020	N501Y, E484K, K417T
Delta	B.1.617.2	India	April 2021	T478K and L452R***
Omicron BA.1	B.1.529.1	South Africa	November 2021	S371L, S373P, S375F, K417N, N440K, **G446S**, S477N, T478K, E484A, Q493R, ***G496S***, Q498R, N501Y, Y505H, **T547K**
Omicron BA.2	B.1.529.2	South Africa	November 2021	S371L, S373P, S375F, T376A, D405N*, R408S K417N, N440K, S477N, T478K, E484A, Q493R, Q498R, N501Y, Y505H
Omicron BA.3	B.1.529.3	South Africa	November 2021	S371L, S373P, S375F, D405N*, K417N, N440K, S477N, T478K, E484A, Q493R, Q498R, N501Y, Y505H
Omicron BA.4 or BA.5	–	South Africa	November 2021	S371L, S373P, S375F, T376A, D405N*, R408S K417N, N440K, S477N, T478K, E484A, L452R***, F486V**, Q493R, Q498R, N501Y, Y505H

*Note*: The first country, date of the first detection, and notable mutations in the receptor binding domain (RBD) of the spike protein were also shown.

D614G mutation is present in all VOCs. Mutations highlighted bold = Omicron BA.1. Mutations underlined = Omicron BA.2, 4, and 5. D405N* is present in BA.2, 3, 4 and 5. F486V** is only present in BA.4 or BA.5 which are identical in their spike proteins. L452R*** is present in Delta, BA.4, or BA.5.

Abbreviations: VOC, variants of concern; WHO, World Health Organization.

Within the last 2 years of becoming pandemic, the VOCs of SARS CoV‐2 have been emerging and out‐competing each other. For example, in England, the wildtype variant of SARS CoV‐2 was first replaced by the Alpha variant, which was later dominated by the Delta variant, and subsequently, the Omicron subvariant BA.2 has been dominant over the other previous variants including BA.1 subvariant.^3^ Additionally, studies reported recombination or simultaneous infections with two variants in the same individual. For instance, XE variants (recombined BA.1/BA.2) or co‐infections (BA.1 and BA.2) were reported in England and North Macedonia.^3^, ^4^ Recently, BA.3, BA.4 and BA.5 sub‐lineages of Omicron are out‐competing with BA.1 and BA.2.^2^, ^5^ This indicates that the virus is continuously acquiring novel mutations that could produce novel emerging variants.

It is worth mentioning that the mutations occurred in amino acids of the spike protein (D614, N501, L452, E484, and K417) have played important roles in evolving VOCs. Among these mutations, all VOCs carry the D614G mutation while the N501Y mutation is only absent in the Delta variant, which has the L452R mutation. Both E484K/A and K417N/T mutations are present in Beta, Gamma, and Omicron. These mutations have had major impacts on the pandemic through increasing transmissibility, infectivity, receptor binding, and the ability of the virus to evade immunity.^6^, ^7^, ^8^

Omicron variant (B.1.1.529 sub‐lineage BA.1), having approximately 50 mutations in the viral genome, 30 of the mutations in the spike gene and 15 of the mutations in the RBD, was detected in more than 50 countries.^9^ Eleven mutations, (K417N, N440K, G446S, S477N, T478K, E484A, Q493R, G496S, Q498R, N501Y, and Y505H), which are present in the RBD of the Omicron variant, have been linked to therapeutic antibody resistance and vaccine evasions.^10^ The resistance of the Omicron BA.1 sub‐variant to the sero‐neutralizations of both vaccinated and recovered adult individuals might have caused this sub‐variant to be dominant in Italy and Europe at the beginning of 2022.^11^

Several countries have conducted molecular, immunological and epidemiological studies about both Omicron and Delta variants. Nonetheless, such kind of studies is seldom in developing countries such as Iraq. For example, in the United States, both Delta (B.1.617.2), and Omicron (B.1.1.529) variants had been dominated as of February 2022.^12^ In Denmark, the rate of transmission and reinfections in Omicron is higher compared to Delta which could be linked to antibody evasions of the Omicron variant, however, the risk of hospitalizations is reduced in the Omicron variant.^13^ Whole genomic surveillance has found both Omicron and Delta variants of SARS CoV‐2 in Armenia that originated from multiple countries and Covid‐19 deaths were higher in Delta than in Omicron.^14^

Several methods have been developed to identify the recently circulated VOCs (e.g., Delta and Omicron), including spike gene target failure,^15^ pyrosequencing of spike gene,^12^ spike gene sequencing,^1^, ^11^ variant‐specific RT‐PCR testing and whole‐genome sequencing.^13^ In Denmark, L452 and 452R markers were putatively used to differentiate between Omicron and Delta variants, respectively.^13^ Identifications of both Delta and Omicron variants, by using either rapid molecular methods or sequencing, have been investigated in many countries including the United States,^16^, ^17^, ^18^, ^19^ United Kingdom,^3^, ^20^ Germany,^21^, ^22^ Denmark,^13^ Italy,^11^, ^23^ Ecuador,^24^ China,^25^ Taiwan,^6^ Belgium,^1^ Malaysia,^26^ Iran,^27^ and Japan.^28^ Such rapid molecular methods are uncommon in Iraq and Kurdistan region.

SARS CoV‐2 genotyping is important to monitor the viral evolutions that lead to immune evasions, increasing transmissibility, infectivity, and severity of Covid‐19 infections. In addition, conducting epidemiological surveillance, immunization programs, testing the therapeutic efficacy of antivirals, and examining reinfections will require prior knowledge about the viral variants. Whole genome sequences, which have been considered the gold standard for identifications of VOCs by the CDC,^29^ are not available in most laboratories, particularly if it is absent in developing countries (e.g., Iraq). Identifications of SARS CoV‐2 VOCs are challenging in such countries since not enough funds or grants have been provided. During preparations of the current manuscript, a study has reported only two cases, a Delta and an Omicron in Iraq, using whole spike gene amplifications and sequences.^30^ This study has used several primer pairs, reactions, and multiple sequence alignments. Therefore, rapid and economic methods are required for the prompt identification of VOCs in such countries as Iraq that help healthcare professionals, virologists and molecular immunologists to monitor the epidemiological aspects of the virus in the region. The objective of the current study is to identify the novel VOCs in Sulaymaniyah city using rapid and cost‐effective methods including mutation‐specific rRT PCR probes, K417N, D614G^31^ and to amplify the partial sequence of the spike gene by conventional RT PCR and Sanger sequencing.

## 2 MATERIALS AND METHODS

### 2.1 Sample collections

The nasopharyngeal samples were collected from the COVID‐19 suspected individuals who visited polyclinics in Sulaymaniyah city from November 2021 to January 2022. The samples were preserved in 3 mL of VTM and directly transferred to the lab. The fresh samples were analyzed for COVID‐19 presence using a real‐time PCR kit (VIASURE), and positive samples were kept in a −80°C freezer for later investigations.

### 2.2 Ethical approval

Consent forms were filled out for each patient and ethical approval (No. 000353) for conducting the current study was obtained from the University of Garmian Ethical Committee which adhered to WHO Guidelines on Ethical Issues in Public Health Surveillance and to the principles of the Declaration of Helsinki.

### 2.3 Plan of the study

**Figure 1:**
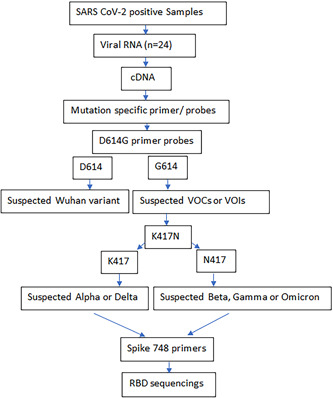
Diagram of current study plan. VOC, variant of concern. VOI, variant of interest.

The study was conducted according to the plan as shown in Figure 1.

### 2.4 Viral RNA extraction

Total RNA was extracted from 200 µL of VTM samples. The extractions were performed by an automated extractor (ZINEXTS; MagPurix‐12A) employing an RNA extraction kit (ZINEXTS). The manufacturer's instructions were followed and the purified RNAs were used directly in investigations or kept in a −80°C freezer for later use.

### 2.5 rRT PCR for SARS CoV‐2 identification

The extracted RNAs were checked for the presence of COVID‐19 nucleic acid using an rRT PCR kit (VIASURE). The manufacturer's instructions were followed. After the rehydration of lyophilized pellets by rehydration buffer supplied by the manufacturer company, the master mix (15 µL) and extracted RNA sample (5 µL) were mixed in a PCR tube. The PCR protocol were as follows: 45°C for 15 min, 95°C for 2 min, and repeated 45 cycles of 95°C for 10 s and 60°C for 50 s.

### 2.6 Complementary DNA (cDNA) synthesis

The cDNAs were synthesized from the extracted RNAs using a cDNA synthesis kit (Addbio). According to the kit instruction, master mixes (10 µL) containing reverse transcriptase, dNTPs, and random hexamer primers were mixed with extracted RNAs (10 µL) and the reaction was performed as follows: 45°C for 60 min, 80°C for 5 min, and 12°C for 10 min. The cDNAs were kept in −20°C freezers for later use.

### 2.7 Identifying D614G and K417N mutations

Real‐time PCR reactions were performed for the identification of D614G and K417N mutations in the spike protein using TaqMan probes. The primers and probes for D614G mutation are designed as it is mentioned in our previous study.^31^ The primers for K417N were designed to amplify a 100 bp region between nucleotides 22769 and 22868 in Wuhan‐Hu‐1, complete genome (GenBank = MN908947.3), and the probes were designed to hybridize to nucleotides between 22795 and 22820. The primers and probes (Table 2) were inspected for melting temperatures, GC contents, lengths, product sizes, and locations using the NCBI database and online IDTDNA qPCR design tool (https://www.idtdna.com). The 20 µL reaction mixture contained 8 µL of cDNA with 8 µL of PCR master mix (HighQU), 1 µL (5 µM) of each forward and reverse primer, and 1 µL (5 µM) of each TaqMan probe (either D‐FAM and G‐HEX or K‐FAM and N‐HEX). The reactions was as follows: initial denaturation at 95°C for 5 min then 45 cycles of denaturation at 95°C for 10 s, annealing/extension at 61°C (D614G) or 65°C (FOR K417N) for 60 s using real‐time PCR machine (BIOER LineGene 9600 plus).

**Table 2:** Primers and probes used in the current study.

Primers/probes	Nucleotide sequences	length	mt°C	GC%	bp
D614G F	GAGATTCTTGACATTACACCATG	23	59	39	169
D614G R	CTGTAGAATAAACACGCCAAG	21	59	43	
D614 D FAM	FAM‐ TTCTTTATCAGG **A** TGTTAACTGCACAG‐ BHQ1	27	64	37	
G614 G HEX	HEX‐ TTCTTTATCAGG **G** TGTTAACTGCACAG‐ BHQ2	27	65	41	
K417N F	AGAGGTGATGAAGTCAGACAAAT	23	55	39	100
K417N R	AAGCTATAACGCAGCCTGTAA	21	56	43	
K417 **K** FAM	FAM‐TCCAGGGCAAACTGGAAA **G** ATTGCTG –BHQ1	26	64	50	
N417 **N** HEX	HEX‐TCCAGGGCAAACTGGAAA **T** ATTGCTG – BHQ2	26	62	46	
Spike 748 F	AGAGGTGATGAAGTCAGACAAAT	23	55	39	748
Spike 748 R	CTATTAAACAGCCTGCACGT	20	60	45	

### 2.8 Conventional RT PCR for spike 748 primers

The cDNA samples (7 µL) were mixed with same amount of RT PCR master mix (Addbio) with the addition of 0.5 µL of each forward and reverse primers.^32^ The reaction was as follows: an initial denaturation at 95°C for 5 min, then 40 cycles as follows: 20 s at 95°C, 30 s at 61°C, and 35 s at 72°C and a final extension at 72°C for 7 min. The PCR products were run on 1.5% agarose gel and the presence of a 748 bp band was considered a successful amplification. Four samples were sent for sequencing (Sanger dideoxy sequencing, Macrogen Co.) and the sequences were submitted to NCBI using Bankit^33^ for further investigation and phylogenetic analysis.

### 2.9 Phylogenetic analysis

The entire genomes of the Wuhan strain, other VOCs, and RBD sequences of some other different isolates in Iraq and abroad were used in phylogenetics and sequence analysis. A total of 17 sequences including 4 sequences from the current study were analyzed. MEGA 11 software was used to cluster the sequences and the MUSCLE method was used for sequence alignment considering the Wuhan strain as a reference genome. The neighbour‐joining method with (−1000) bootstrap was used to infer the evolutionary history of the isolates.

## 3 RESULTS

### 3.1 Rapid inexpensive methods for identifying D614G and K417N mutations

Positive samples with high Ct values were considered for further analysis and identification of D614G and K417N mutations. The SARS CoV‐2 positive samples (*n* = 24) were exploited by using rRT PCR TaqMan probes designed for identifications of two important mutations including D614G and K417N. The results showed that all 24 samples were G614 mutant. The K417 wildtype is present in the Delta variant while the N417 mutant is present in the Omicron variant (As shown in Figure S1). Therefore, in the current study, rRT PCR primers/probes were developed to distinguish K417 from N417 mutation. As a result of optimizations, it was shown that the mutations can be distinguished at the annealing/extension temperature of 65°C. Out of 24 samples, 3 samples were K417 wildtypes whereas 19 samples were N417 mutants. Interestingly, two samples carried both K417 wildtype and N417 mutant that suggested co‐infections with two different variants, potentially Omicron and Delta.

### 3.2 Identifications of SARS CoV‐2 RBD mutations by DNA sequencings

**Figure 2:**
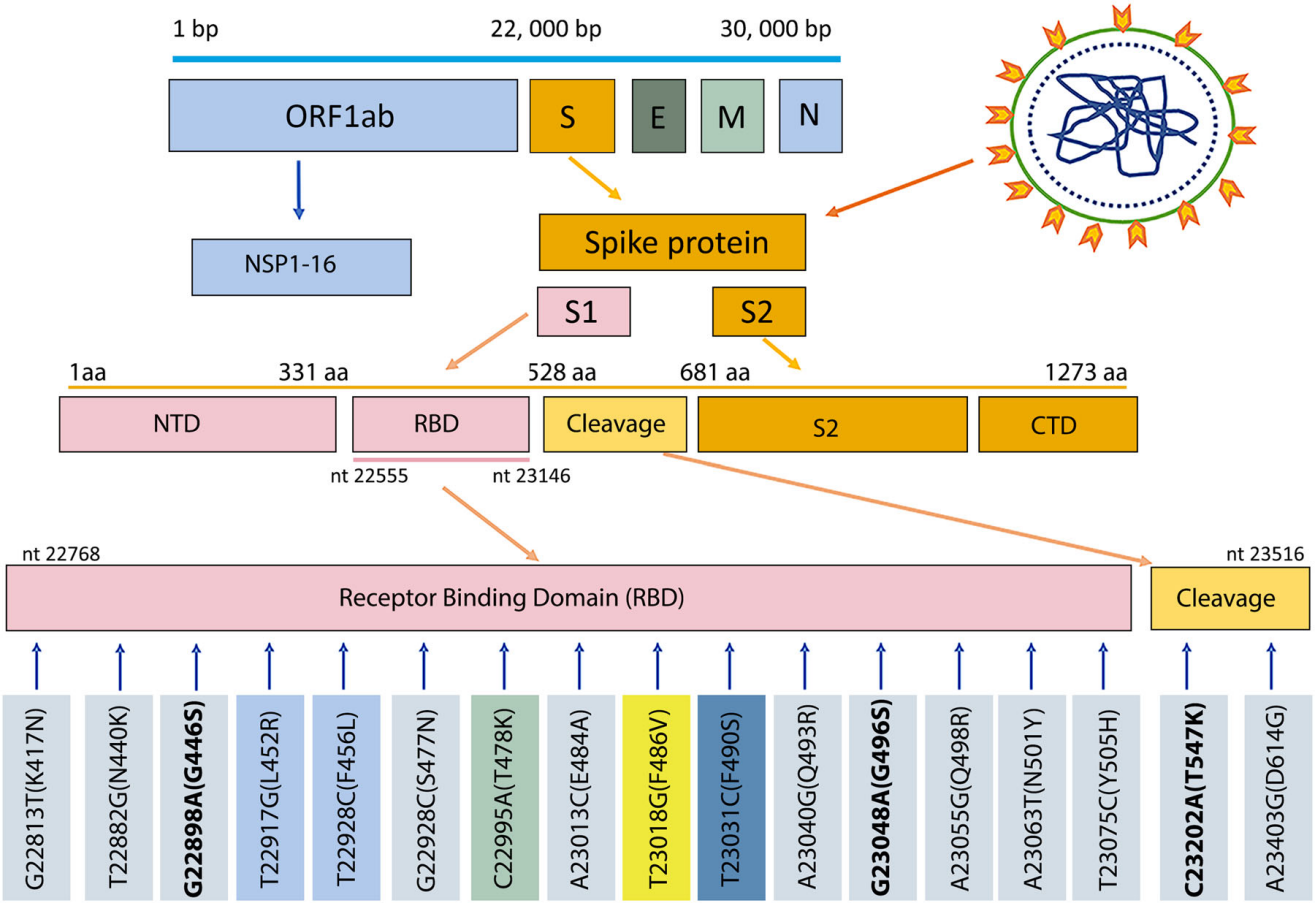
Diagram of SARS CoV‐2 mutations in the receptor binding proteins. ORF1 = Open reading frame. S = Spike protein. E = envelope protein. M = Membrane protein. N = Nuclear protein. NSP = nonstructural protein. S1 = spike protein 1. S2 = Spike protein 2. NTD = N‐terminal domain. RBD = Receptor Binding Domain. Cleavage = cleavage site between S1 and S2. CTD = C‐terminal domain. bp = base pair of nucleotides. aa = amino acid. SARS CoV‐2 has about 30,000 bp. Spike protein has 1273 amino acids. RBD = nucleotides (nt) 22555–23146. The Spike 748 primers used in the current study amplify a region of 748 bp (22768–23516). Mutations were pointed by the arrows. The mutations highlighted grey = Omicron variant. The two mutations highlighted light blue = Delta variant. The mutation highlighted turquoise = Lamda variant. The mutation highlighted light green = Both Omicron and Delta variants. Mutations (highlighted in bold) are present in BA.1 lineage but not in BA.2, G22898A (G446S), G23048A (G496S), and C23202A (T547K). T23018G (F486V) highlighted yellow is present in BA.4 or BA.5. Three mutations in the cleavage site were not detected using these primers: C23525T (H655Y), T23599G (N679K), and C23604A (P681H). Three more mutations in RBD, S371L, S373P, and S375F were not detected by the primers. SARS CoV‐2, severe acute respiratory syndrome coronavirus 2.

After distinguishing the mutations by rapid methods, four samples of different mutants were subjected to conventional PCR using spike 748 primers. The primers can amplify a 748 bp in the RBD region which includes 17 VOC mutations (Figure 2). The PCR products were run on 1.5% agarose gel (As shown in Figure S2) and the remaining PCR products were sent for sequencing (Sanger dideoxy sequencing, Macrogen Co.). The sequencing results were checked and submitted to NCBI under GenBank IDs (ON394474‐ON394477).

As shown in Figure 2, the RBD of SARS CoV‐2 Wuhan strain starts from amino acid 331 to 528,^34^ while the spike 748 primers used in the current study can amplify a region of RBD and cleavage site between S1 and S2 that includes most notable mutations have occurred in the spike protein of the SARS COV‐2 VOCs. Our primers cover 22,768 to 23,516 nucleotides that contain 17 mutations highlighted from K417N to D614G (Figure 2). The sequencing results suggested that SARS CoV‐2 Omicron BA.1 subvariant (GenBanks: ON394475.1, ON394476.1, ON394477.1) which had 13 mutations (K417N, N440K, G446S, S477N, T478K, E484A, Q493R, G496S, Q498R, N501Y, Y505H, T547K, D614G) and a novel Delta variant (GenBank ON394474.1) which had two mutations (T478K and L452R) that specific to Delta variant and two extra novel mutations (F456L and F490S) which are not usually seen in the Delta variant, were circulating in Sulaymaniyah province.

**Figure 3:**
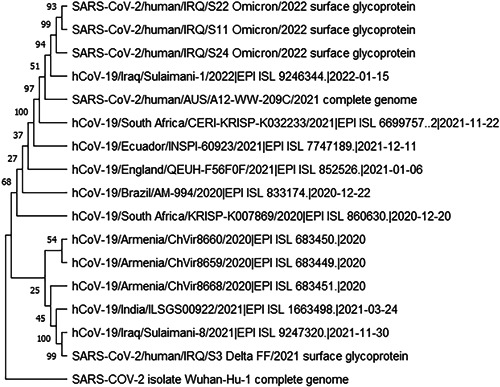
Phylogenetic tree of 17 SARS COV‐2 VOCs (spike protein and whole genome) sequences including four partial spike sequences from the current study. SARS COV‐2, severe acute respiratory syndrome coronavirus 2; VOC, variants of concern.

Our spike 748 primers can discriminate against BA.1 from BA.2/BA.3 and BA.4/BA.4 since mutations (G446S, G496S, and T547K) are only present in BA.1 but absent in BA.2/BA.3, and BA.4/BA.5. Moreover, the mutations (L452R and F486V) are present in BA.4/BA.5 but absent in BA.1, BA.2/BA.3. However, the primers cannot discriminate against BA.2 from BA.3. Additionally, they cannot discriminate against BA.4 from BA.5 because the variants have the same mutations from K417N to D614G.

### 3.3 Phylogenetic analysis

Neighbor‐joining method tree was constructed using 17 full genome and spike sequences with 4 sequences from the current study (Figure 3). Out of 4 analyzed sequences; S11, S22 Omicron variants then the S24 Omicron variant were clustering with a recently identified omicron variant isolated in Sulaymaniyah/Iraq, South African Omicron variant and an Omicron variant isolated in Australia. However, the fourth sequence which is a delta variant S3 is clustering with a recently identified delta variant in Sulaymaniyah/Iraq and the original delta reference variant identified in India.

## 4 DISCUSSIONS

This study exploited both mutation (D614G and K417N) specific RT PCR methods followed by spike 748 sequencings targeting RBD of SARS CoV‐2 spike gene. The results showed that both Delta and Omicron variants had been circulated in Sulaymaniyah city from November 2021 to January 2022. Up to our best knowledge, based on searching in PubMed, during writing this manuscript (24/06/2022), the spike gene of only two cases for each Delta and Omicron variants have been phylogenetically analyzed in Sulaymaniyah province.^30^ Previously, the Alpha variant was potentially identified using N501Y mutation‐specific probes and DNA sequences^35^ that might play roles in SARS CoV‐2 re‐infections in the region.^32^

The current study has found that both Omicron and Delta variants had been spreading among people in Sulaymaniyah city between the end of 2021 and the beginning of 2022. Studies in other countries showed that Omicron variants have dominated Delta variants. Particularly, in China and South Africa, BA.4 and BA.5 sub‐lineages of Omicron have dominated over BA.1.^2^, ^5^ The current study suggested that only BA.1 variant was present in the region. Further study with large epidemiological data is required to investigate the most prevalent variants in the region. Additionally, our study showed that the 2 samples, which carried both K417 wildtype and N417 mutant, are possibly due to co‐infections with both Omicron and Delta variants as previously shown in other countries like England or North Macedonia where both BA.1/BA.2 subvariants were reported in the same individuals.^3^, ^4^ Furthermore, Delta variant usually contains two mutations (T478K and L452R). Nonetheless, two more novel mutations (F456L and F490S) which are not usually seen in the Delta variant, were found in the current study that suggested a novel Delta subvariant had been existing in the region during 2021–2022.

Rapid molecular tests used to screen epidemiological and genomic surveillance of emerging SARS CoV‐2 VOCs have been advantageous over whole genome sequencing in terms of costs, laboratory facilities, proficiencies, and time. Several studies have validated rapid assays developed for VOC identifications. However, the rapid identification of the VOCs has been challenging as a result of mutations that happen in the spike protein gene. In the UK, a study validated reverse transcription recombinase polymerase amplification (RT‐RPA) assays targeting both E and RdRP genes intended to detect VOCs (Alpha, Beta, Delta, and Omicron).^20^ In Italy, the failure of diagnostic kits to target the SARS CoV‐2 spike gene was used for rapid and economic screening of the virus's variants including Delta and Omicron.^23^ In China, a study showed that the mutations that occurred in VOCs (Beta, Gamma, and Delta) have a negative impact on the sensitivity of commercial diagnostic kits due to mismatches located in the primer/probe nucleotide sequences, however, both Alpha and Omicron have no substantial effects.^25^ The efficacies of the previous rapid methods were reduced or failed to identify the K417N, N440K, G446S, and N501Y mutations in the Omicron variant due to intra‐ primer or ‐probe mutations such as Q493R, G496S, Q498R, or Y505H; this makes the diagnostic assays be regularly updated.^21^ Interestingly, no mutations have occurred in our primer/probe binding sites (D614G, K417N, and spike 748) that would mismatch with the SARS CoV‐2 VOCs. Therefore, these primer/probe regions might be considered conserved regions for designing future diagnostic kits. However, our previous primer/probes designed for the N501Y mutation^35^ acquired several mutations that discourage us to use them in the current study. Further methods should be developed (e.g., melting curve analysis) to identify N501Y mutation since there are several mutations around the nucleotide sequences of this mutation that prevent designing N501Y‐specific probes. It is worth mentioning that our primer/probes were used in two different laboratories using different reagents and thermocyclers and the results were similar in this study. In Taiwan, multiplex RT‐PCR with mutation‐specific probes (e.g., K417N, L452R, E484K, E484Q, and N501Y) was used to target the RBD of the spike protein gene to explore the five SARS CoV‐2 VOCs.^6^ In the USA, a molecular beacon was used to target the important mutations such as K417N, L452R, E484K/Q, T478K, and N501Y identifying all VOCs including Delta and Omicron, however, miscellaneous mutations (e.g., L452Q) and mutations as E484K were found in non‐VOCs.^18^ Therefore, sequencing of RBD or genotyping assays^15^ should often require to confirm the rapid test results for screening current or future emerging variants. The K417N probes used in the current study can potentially discriminate between Delta and Omicron variants as concluded to identify the Omicron BA.1 variant.^15^ Similarly, a previous genotyping assay that designed probes to identify a single mutation (e.g., T547K or G399D) was used to discriminate between Alpha, Delta, and Omicron variants.^36^ The current study confirmed the mutations present in the RBD of the spike protein using the sequencings amplified by 748 primers, which can identify notable mutations from K417 to D614 amino acids of the spike protein that are present in almost all VOCs. Both Delta (B.1.617.2‐AY.43) and Omicron (B.1.1.529 BA.1) variants were identified in Italian children, using primers amplifying the RBD and sequence analysis.^11^

Sequencing of the SARS CoV‐2 spike gene has been used to identify the most common mutations. For instance, in Iran, nested PCR and sequencing were used to amplify the RBD of the spike protein that has found mutations (D614G, L452R, and T478K) in the Delta variant, and (D614G, T478K, G496S, and T547K) in Omicron variant.^27^ Furthermore, in Japan and Iraq studies sequenced the whole spike protein gene using several pairs of primers and PCR reactions that identified mutations of VOCs.^28^, ^30^ In the current study, 13 mutations (K417N, N440K, G446S, S477N, T478K, E484A, Q493R, G496S, Q498R, N501Y, Y505H, T547K, and D614G) in the Omicron BA.1 subvariants and five mutations (D614G, L452R, T478K, F456L, F490S) in the novel Delta variant were found using only a pair of primer and a single reaction that is less expensive and more time‐saving than previous methods. Interestingly, the F490S mutation found in our novel Delta variant has also been seen in Lambda variant B.1.1.1 and a Delta sublineage in Italy.^37^ The F456L mutation found in our Delta variant has also been found in Delta sublineage AY.122.^22^ Both F490S and F456L detected in the current study were not found in the previous study performed in Iraq.^30^ Therefore, further study is needed to screen the novel Delta variant reported in the current study. This novel variant can be considered for whole genome sequencing. In Belgium, all VOCs including the omicron variant were identified by sequencing of the RBD using PCR primers amplified a 733 bp region of the spike protein from amino acid AA360 to AA588 that were validated by whole genome sequencings with GISAID (accession number EPI_ISL_6794907).^1^ This case in Belgium was the first report of the omicron variant on 25 November 2021 in Europe with GenBank accession number OL672836.^38^ A blind panel study in the Uniteed States showed that the results of rapid RT PCR corresponded with those of the whole genome sequences for identifications of mutations (e.g., N501Y, E484K, S982A, P681R, and L452Q) in the spike protein of VOC and variants being monitoring, however, some inadequate results were seen in some Alpha, Omicron and Delta variant samples due to expected mutations, contaminations or RNA degradations.^17^ Mutations in variant‐specific probes impact the efficiency of RT PCR diagnostic techniques.^16^, ^26^ Therefore, both rapid probe‐specific assays and spike gene sequencing are required to monitor the viral evolutions and emergence of novel variants.^19^

Phylogenetic analysis of four sequences (3 Omicron and 1 Delta) from the current study samples revealed very a close relation of our Omicron variants to the Omicron variant which has previously sequenced in the same region by Rashid and Salih,^30^ suggesting a similar source of Omicron variant circulating in Sulaymaniyah province. However, despite the presence of two unique mutations in our Delta variant but it was still clustering with the Delta variant recorded by the same researchers in Sulaymaniyah, suggesting the probability of divergence of our Delta variant from the circulating Delta variant in the region.

The current study has limitations as the mutation‐specific probes can only target D614G and K417N. Additionally, the primers used can only amplify a sequence fragment of the spike protein. However, the D614G primer probes can be used for discrimination of the original D614 variant, from the mutant variant G614. The D614 bearing variant such as A.27 and A.29 have been detected in Germany,^22^ while the G614 mutation is prevalent in all VOCs.

Overall using mutation‐specific primer/probes together with RBD sequencings can be used as reliable methods for rapid epidemiological screening of SARS CoV‐2 VOCs that can support hospitals in developing countries to apply both vaccination and therapeutic strategy.

## 5 CONCLUSIONS

By exploiting rapid, less expensive methods such as TaqMan probes and partially sequenced spike gene of SARS CoV‐2, the current study suggested that both Omicron BA.1 and novel Delta variants are present in Sulaymaniyah province, Iraq. Further investigation should focus on surveillance of both past and future VOCs using the aforementioned methods.

## AUTHOR CONTRIBUTIONS

*Conceptualization*: Mariwan Kadir Rasheed, Harem Abdalla Awrahman, Sirwan M. Amin Al‐Jaf, Sherko S. Niranji. *Planning*: Mariwan Kadir Rasheed, Harem Abdalla Awrahman, Sirwan M. Amin Al‐Jaf, Sherko S. Niranji. *Sample collections*: Mariwan Kadir Rasheed, Harem Abdalla Awrahman. *PCR works*: Mariwan Kadir Rasheed, Harem Abdalla Awrahman. *Primer and probes design*: Sherko S. Niranji, Sirwan M. Amin Al‐Jaf. *Figure designs*: Sherko S. Niranji and Sirwan M. Amin Al‐Jaf. *Drafting the article*: Sherko S. Niranji, Sirwan M. Amin Al‐Jaf. *Editing manuscript*: Mariwan Kadir Rasheed, Harem Abdalla Awrahman, Sirwan M. Amin Al‐Jaf, Sherko S. Niranji.

## CONFLICT OF INTEREST STATEMENT

The authors declare no conflicts of interest.

## Supporting information

Supporting Information.

## Data Availability

All data are available through the corresponding author Sherko S. Niranji. The sequences are available in NCBI GenBanks: ON394475.1, ON394476.1, ON394477.1, and ON394474.1.
