# Supplementary material for: Identification of SARS CoV‐2 Omicron BA.1 and a novel Delta lineage by rapid methods and partial spike protein sequences in Sulaymaniyah Province, Iraq
Source: Immun Inflamm Dis. 2023 Mar 17;11(3):e801. doi: 10.1002/iid3.801 (PMC10022420; doi:10.1002/iid3.801)
Supplement: Supplementary file 1 — Supporting Information. [file IID3-11-e801-s001.pdf]

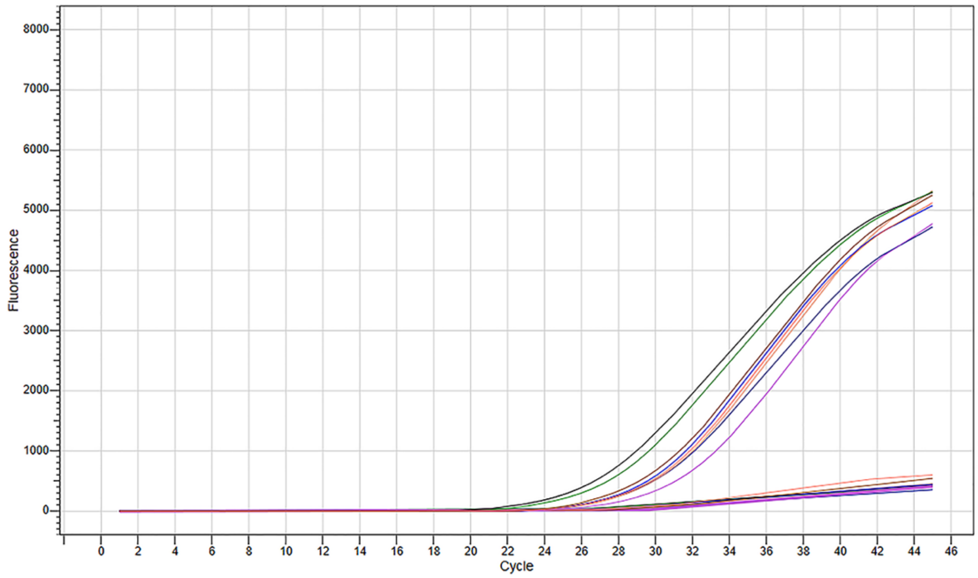

Figure S1: Raw data of Real-time rtPCR of K417N mutation for some the studied samples (8 samples).

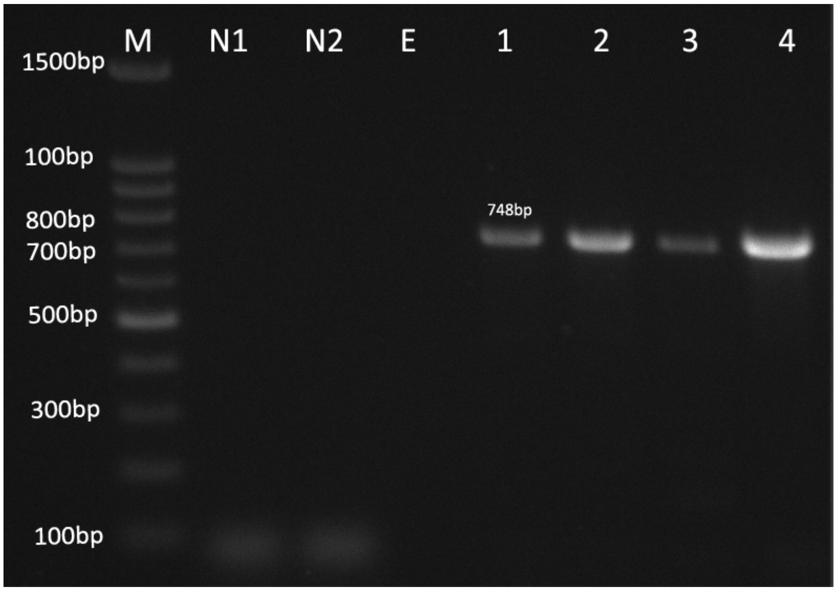

Figure S2: Agarose gel image of conventional PCR M= 100 DNA markers. N1 and N2= negative controls that contain no DNA. E= empty well. 1,2,3,4= positive samples amplified using spike 748 F and spike 748 R primers. Appearance of a 748bp band indicates the amplification of a 748bp region from spike gene of SARS-COV-2. This region is located between the nucleotides 22768 to 23516 of the genome. These samples were sequenced and used in phylogenetic analysis.
